# U-SMILE: a brief version of the Short Multidimensional Inventory on Lifestyle Evaluation

**DOI:** 10.47626/2237-6089-2023-0722

**Published:** 2025-04-07

**Authors:** Raquel B. De Boni, Jurema C. Mota, Felipe Barreto Schuch, Daniel Alvarez Pires, Thiago Sousa Matias, Renato Sobral Monteiro-Junior, Andrea C. Deslandes, Danilo R. Silva, Helena Ferreira Moura, Nicole Galvão-Coelho, Fabianna Resende de Jesus-Moraleida, Flavio Kapciznki, Vicent Balanzá-Martinez

**Affiliations:** 1 Instituto de Comunicação e Informação Científica e Tecnológica em Saúde Fundação Oswaldo Cruz Rio de Janeiro RJ Brazil Instituto de Comunicação e Informação Científica e Tecnológica em Saúde (ICICT), Fundação Oswaldo Cruz (FIOCRUZ), Rio de Janeiro, RJ, Brazil.; 2 Universidade Federal de Santa Maria Departamento de Métodos e Técnicas Desportivas Santa Maria RS Brazil Departamento de Métodos e Técnicas Desportivas, Universidade Federal de Santa Maria, Santa Maria, RS, Brazil.; 3 Universidade Federal do Rio de Janeiro Instituto de Psiquiatria Rio de Janeiro RJ Brazil Instituto de Psiquiatria, Universidade Federal do Rio de Janeiro, Rio de Janeiro, RJ, Brazil.; 4 Universidade Federal do Pará Castanhal PA Brazil Programa de Pós-Graduação em Ciências do Movimento Humano, Universidade Federal do Pará, Castanhal, PA, Brazil.; 5 Universidade Federal de Santa Catarina Departamento de Educação Física Florianópolis SC Brazil Departamento de Educação Física, Universidade Federal de Santa Catarina, Florianópolis, SC, Brazil.; 6 Universidade Estadual de Montes Claros Departamento de Educação Física Montes Claros MG Brazil Programa de Pós-Graduação em Ciências da Saúde, Departamento de Educação Física, Universidade Estadual de Montes Claros (Unimontes), Montes Claros, MG, Brazil.; 7 Unimontes Grupo de Estudos e Pesquisas em Neurociência, Exercício, Saúde e Esporte Montes Claros MG Brazil Grupo de Estudos e Pesquisas em Neurociência, Exercício, Saúde e Esporte, Unimontes, Montes Claros, MG, Brazil.; 8 Universidade Federal de Sergipe Departamento de Educação Física São Cristóvão SE Brazil Departamento de Educação Física, Universidade Federal de Sergipe, São Cristóvão, SE, Brazil.; 9 Universidade de Brasília Faculdade de Medicina Brasília DF Brazil Faculdade de Medicina, Universidade de Brasília, Brasília, DF, Brazil.; 10 Universidade Federal do Rio Grande do Norte Departamento de Fisiologia e Comportamento Natal RN Brazil Departamento de Fisiologia e Comportamento, Universidade Federal do Rio Grande do Norte, Natal, RN, Brazil.; 11 Universidade Federal do Ceará Departamento de Fisioterapia Programa de Pós-Graduação em Fisioterapia e Funcionalidade Fortaleza CE Brazil Programa de Pós-Graduação em Fisioterapia e Funcionalidade, Departamento de Fisioterapia, Universidade Federal do Ceará, Fortaleza, CE, Brazil.; 12 Universidade Federal do Rio Grande do Sul Instituto Nacional de Ciência e Tecnologia Translacional em Medicina Porto Alegre RS Brazil Instituto Nacional de Ciência e Tecnologia Translacional em Medicina (INCT-TM), Universidade Federal do Rio Grande do Sul (UFRGS), Porto Alegre, RS, Brazil.; 13 UFRGS Hospital de Clínicas de Porto Alegre Departamento de Psiquiatria Porto Alegre RS Brazil Laboratório de Psiquiatria Molecular, Departamento de Psiquiatria, Hospital de Clínicas de Porto Alegre (HCPA), UFRGS, Porto Alegre, RS, Brazil.; 14 University of Valencia Centre for Biomedical Research in Mental Health Network Department of Medicine Valencia Spain Teaching Unit of Psychiatry and Psychological Medicine, Department of Medicine, Centre for Biomedical Research in Mental Health Network (CIBERSAM), University of Valencia, Valencia, Spain.

**Keywords:** Lifestyle, health questionnaires, validation, university students

## Abstract

**Objective:**

Lifestyle Medicine comprises six domains: diet, substance use, physical activity, stress management, social connection, and sleep. The comprehensive assessment of lifestyle is challenging, but the Short Multidimensional Inventory on Lifestyle Evaluation (SMILE) was developed to fill out this gap. In this paper, we describe the development and the psychometric properties (internal consistency, concurrent and convergent validity) of a shorter version of the SMILE among university students.

**Methods:**

Data from a cross-sectional study including 369 students from 10 Brazilian universities were used. Considering a theoretical nomological net, we performed exploratory factor analysis (EFA) to obtain the most parsimonious, interpretable, and good-fitting model.

**Results:**

The final model was called U-SMILE, comprised 24 items, and presented acceptable internal consistency (Cronbach's α = 0.73, McDonald's ω = 0.79). To evaluate the concurrent validity of the U-SMILE, we compared it to the original SMILE and found a high correlation between the instruments (Spearman's r = 0.94). Furthermore, we evaluated convergent validity by examining the U-SMILE correlation with the Patient Health Questionnaire (PHQ-9) (Spearman's r = −0.517), and Generalized Anxiety Disorder Questionnaire (GAD-7) (Spearman's r = −0.356), two validated instruments to screen for depression and anxiety, respectively.

**Conclusion:**

Our findings suggest that the U-SMILE is a valid instrument for assessing lifestyle among university students. We recommend that the use of U-SMILE to evaluate overall lifestyle scores rather than individual domain scores. Finally, we discuss the importance of clarifying the definitions of lifestyle and related constructs in future research.

## Introduction

Unhealthy lifestyle behaviors are major risk factors for morbidity and mortality worldwide.^1,2^ Those behaviors are unlikely to occur in isolation but instead tend to cluster together among individuals.^3^ Clusters with a higher number of unhealthier behaviors are associated with a reduced survival time without disability and higher mortality compared to these behaviors in isolation.^4-7^

In the last decades, Lifestyle Medicine (LM) emerged as a branch of evidence-based medicine to deliver strategies for changing unhealthy behaviors to prevent and treat chronic diseases, including mental health disorders.^8^ The European Lifestyle Medicine Organization (ELMO)^9^ defined LM as "an inter-disciplinary field of internal medicine, psychosocial and neurosciences, public and environmental health, and biology. Key LM principles include prevention strategies that address lifestyle habits, the underlying biological causes and the pathophysiology common to lifestyle-related diseases (e.g. low-grade systemic inflammation, dysregulated stress axis, metabolic dysfunctions, etc."

The American College of Lifestyle Medicine (ACLM)^10^ proposes that the six main pillars of LM are diet, substance use, physical activity, stress management, social connection, and sleep. Although the LM definition and the target areas for interventions have been discussed in the last years, the concept of "lifestyle" is still under debate and may be hard to operationalize.^11^ As such, questionnaires assessing multiple lifestyle behaviors may consider different domains/dimensions. For instance, two widely used questionnaires for evaluating multiple lifestyle behaviors are the Fantastic Lifestyle Checklist^12^ and the General Lifestyle Questionnaire (GLQ).^13^ The Fantastic Lifestyle Checklist assesses nine domains, namely family and friends, physical activity, nutrition, tobacco and toxics, alcohol intake, sleep, seat belt use, stress, safe sex behavior patterns, insight, and career. On the other hand, the GLQ evaluates five domains: physical, cognitive, social, and other leisure activities, sleep, food, tobacco, and alcohol consumption. It is worth noting that the two questionnaires do not evaluate the same lifestyle domains, and none of them follows exactly the same domains proposed by the ACLM and ELMO.

**Figure 1:**
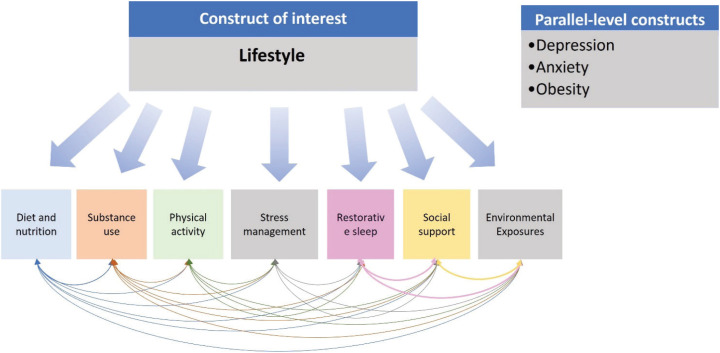
Hypothetical nomological network of the lifestyle construct.

At the same time, there are numerous questionnaires available to measure a lifestyle domain isolated (i.e., questionnaires to evaluate alcohol use, or physical activity, or diet and others). However, to perform a comprehensive assessment of lifestyle by adopting multiple questionnaires may increase the burden for research participants and research costs. Furthermore, lifestyle behaviors are evaluated as independent risk factors and disregard the clustering and interconnection of behaviors.^14,15^ To overcome these barriers, the Short Multidimensional Inventory on Lifestyle Evaluation (SMILE), a 43-item questionnaire, was developed to evaluate the six lifestyle domains proposed by the ACLM and, additionally, environmental exposures.^16^ The development of the SMILE followed a multiple step process that included reviewing lifestyle questionnaires, expert's feedback and revisions, and face validity as described elsewhere.^16^ The hypothetical nomological network of the lifestyle construct, as well as the hypotheses surrounding convergent validity and factor structure of the SMILE are presented in Figure 1. It was expected that lifestyle domains presented correlations among each other due to the clustering of healthy/unhealthy behaviors. Furthermore, worse lifestyle scores should be associated with depression, anxiety, and obesity (among other health outcomes).

In 2020, during the beginning of the coronavirus disease 2109 (COVID-19) pandemic, a shorter version of the SMILE was developed and had its initial psychometric characteristics evaluated.^16,17^ Such development was necessary due to the changes imposed by social distance and confinement essential to control virus’ dissemination. In this process, some questions were dropped because they were not appropriate for the lockdown/confinement, resulting in the SMILE-C (SMILE for confinement) questionnaire. This shorter version comprised 27 items and presented good initial psychometric properties. However, almost 3 years later, individuals are reassuming their previous behaviors insofar in-person activities were restored in most of the countries.

This interplay between social isolation and lifestyle prompts us to pay attention to contexts in which lifestyle can be dynamic and influenced.^18^ For example, university years impose shifts in social, academic, and financial demands to students,^19,20^ and adjustments to these new demands may impact their lifestyle.^21^ It is well documented, for instance, that university students often present poor and unbalanced diet, high levels of physical inactivity, and sedentary behavior, high rates of alcohol and other substance use, poor sleep quality, and high screen time.^22-25^ Although these pieces of evidence demonstrate that university students present poor lifestyle behavior, the extant evidence relies on questionnaires that evaluate a single domain in isolation.

Therefore, considering that certain questions in the SMILE, which were deemed irrelevant in the context of the pandemic, may now hold relevance in the post-COVID society, and recognizing the necessity for a concise multidimensional lifestyle scale that specifically addresses the pertinent domains for university students, this paper endeavors to outline the development process of a short version of the SMILE for university students. Furthermore, it aims to present the initial psychometric properties of this scale (internal consistency, convergent, and concurrent validity)

## Methods

Data from a cross-sectional study conducted in 10 Brazilian universities (covering nine states and the five Brazilian macro-regions) were used. Data collection was conducted online using a questionnaire developed in Research Electronic Data Capture (REDCap^®^). The assessments took place between May-December 2022.

### Study population

A convenience sample of students was recruited using online resources such as advertising on social media, the official university website, and direct emails. Newsletters and posters were fixed on the university walls with the link/QR code to the study survey. Further face-to-face invitations through flyers distribution to students gathering places such as university restaurants, parks, and lectures.

Inclusion criteria were 1) being 18-35 years old and 2) having read and agreed to the consent form. Participants with missing data on the SMILE were excluded from the analysis, but not other exclusion criteria was adopted.

### Sample size

A sample size above 300 individuals is considered large enough to conduct an exploratory factor analysis (EFA), as revised by Boateng et al.^26^ In the present study 369 questionnaires were responded, thus reaching a suitable sample size for the aimed purpose.

### Measures and assessments

The survey included questions on lifestyle, mental health symptoms, and demographics. Lifestyle was assessed using the SMILE. The questionnaire included the 43-item SMILE questionnaire, which is self-reported and has been previously validated for online use. Responses are provided through a four-item Likert scale (Always, Often, Seldom, Never) and scores are calculated by adding up all the answers. The higher the score, the better the lifestyle.^16^

Mental health problems were assessed at two levels. At the first level, the Diagnostic and Statistical Manual of Mental Disorders, 5th edition (DSM-5) Level 1 Cross-Cutting Symptom Measure for Adults^27^ will be answered by all participants. The DSM-5 Level 1 Cross-Cutting Symptom Measure is a self-reported questionnaire that assesses important domains across most psychiatric diagnoses. The adult version is composed of 23 questions that assess 13 psychiatric domains: depression, anger, mania, anxiety, somatic symptoms, suicidal ideation, psychosis, sleep problems, memory problems, repetitive thoughts and behaviors, dissociation, personality functioning, and substance use. This is a 5-point Likert scale in which participants will respond "how much (or how often) you have been bothered by" a given problem during the past 2 weeks. Responses range from 0 = None (not at all) to 4 = Severe (nearly every day). Individuals presenting scores equal or greater than 2 for depression and anxiety symptoms, subsequently answered the Patient Health Questionnaire^28^ (PHQ-9) and the Generalized Anxiety Disorder Questionnaire (GAD-7). The PHQ-9 is a 9-item questionnaire for screening major depression. The scores range from 0 to 27, and scores ≥ 9 indicate a positive screening for depression. The PHQ-9 is widely used and was previously validated in Brazil.^29^ The GAD-7 is a 7-item questionnaire used for the screening of generalized anxiety disorder,^30^ validated in Brazil with cut-off ≥ 10.^31^

Sociodemographics included sex, age and body mass index (BMI). BMI was measured through self-reported anthropometric measurements "What's your height (cm)?" and "What is your weight (kg)?" Subsequently, the BMI variable was calculated as body weight in kg divided by height in squared meters and categorized according to the World Health Organization (WHO) criteria as low weight/normal (≤ 24.9 kg/m²), overweight (25.0-29.9 kg/m²), and obesity (over 30 kg/m²).

### Statistical analysis

Initially, the specificity and redundancy of the 43 SMILE's items were evaluated among the authors because nonspecific items may affect the factor structure, and redundancy may affect convergent validity (25). Two Social Support's items were deemed to be non-specific (i.e., "Do you enjoy your leisure time?" and "Are you satisfied with your sexual life?"), and one item was considered redundant ("Do you take part in celebrations/reunions with family/friends/colleagues?"). Those items were excluded from subsequent analysis.

All the remaining 40 items were checked for normality using the Kolmogorov-Smirnov test (Supplementary Material S1). Due to non-normality, the correlation of items was evaluated through a polychoric matrix. Three items that did not present a ≥ 0.300 correlation with any other items were excluded (D-I1, E_I22, S_I27) (see Supplementary Material S2, available as CSV file for download).

Afterwards, an EFA was performed including the 37 items. This first model was estimated without *a priori* specifications about the number of factors. Principal axis factoring (PAF) was used for factor extraction, using the eigenvalues to determine the number of factors and Oblimin with Kaiser normalization for matrix rotation. Items were then eliminated if 1) presented loading < 0.30 in all factors, 2) presented cross-loading with similar magnitude in two or more factors, and 3) presented higher load in factors different than defined in the hypothesized nomological net (Figure 1). Model fit was evaluated through the root mean square error of approximation (RMSEA), and Tucker-Lewis index (TLI). RMSEA values lower than 0.08, and TLI values above 0.90 were considered acceptable. The solution was critically evaluated within the context of the questionnaire hypothesized structured (Figure 1). This process was repeated until the most parsimonious, interpretable, and good-fitting solution was obtained (called U-SMILE).

Internal consistency was evaluated using Cronbach's α (which was considered acceptable if ≥ 0.70) and McDonald's ω (acceptable if > 0.60). The criterion validity and construct validity were evaluated through concurrent validity and convergent validity, respectively (26). Concurrent validity (i.e., comparison with the reference standard) was evaluated by analyzing the correlation of the U-SMILE with the SMILE. The Spearman's rank correlation coefficient was used because data did not show normal distribution in the Kolmogorov-Smirnov (KS) tests. Convergent validity (comparison with other measure that is a related, but a different construct) was evaluated by analyzing the correlation of the U-SMILE with PHQ-9, GAD-7, and BMI (Spearman's rank correlation coefficient).

Finally, the mean U-SMILE scores were compared between individuals with/without positive screenings for depression and anxiety, and with low/normal BMI vs. obesity. The comparisons were tested using the Mann Whitney's test at significance level of 5% because data did not show normal distribution in the KS tests. All the analyses were conducted in SPPS 20.0 and open-source software R 4.3.2.

### Ethical considerations

The study was approved by all participating ethics committee study sites under register # 55481422.5.1001.5346. All participants read and consent to participate in the study.

## Results

A total of 369 students filled in the SMILE (58.5% women) and were included in the present analysis**.** Of those, 34.7% presented a positive screening for depression (PHQ-9 ≥ 9), 42.8% presented a positive screening for anxiety (GAD-7 ≥ 10), and 9% presented BMI higher than 30.

In the first EFA model, we found an 11-factor solution (Table 1). This model was interpreted following the nomological net. Factor 1 was considered to be measuring a different construct, i.e., well-being, and the items E_I20, E_I23, E_I24 and SS_I36 were dropped. Item E_I19 (Practice a faith or religion) was kept because it may be considered a strategy to deal with stress. Additionally, item AF_I14 was dropped because did not present a load higher than 0.3 in any factor.

**Table 1:** Results from the exploratory factorial analysis EFA for reaching the first solution for reducing the SMILE (n = 369), Brazil, 2022

		Factor										
Domain/Item		1	2	3	4	5	6	7	8	9	10	11
Diet												
	D_I2 Check labels	-0.053	0.056	-0.075	-0.008	-0.008	0.190	-0.001	-0.065	0.169	-0.011	**-0.451**
	D_I3 Eat processed food	-0.124	0.097	-0.094	-0.001	0.031	-0.032	**-0.921**	-0.067	-0.090	0.044	0.076
	D_I4 Eat fast-food when sad	0.028	0.080	0.113	-0.126	-0.044	0.182	**-0.362**	0.049	0.058	0.037	-0.033
	D_I5 Eat healthy foods	-0.004	0.030	-0.097	0.041	-0.113	0.034	-0.015	-0.244	0.078	-0.017	**-0.454**
	D_I6 Keep meal schedule	0.128	-0.035	0.259	0.106	**-0.364**	0.008	-0.115	0.127	0.041	0.226	**-0.382**
	D_I7 Share main meals	0.058	-0.037	-0.007	0.051	-0.155	0.097	0.008	-0.144	-0.096	**0.511**	0.066
											
Substance use												
	S_I8 Binge drinking	0.116	**0.541**	0.215	-0.130	0.076	0.126	-0.053	0.131	-0.017	0.090	-0.113
	S_I9 Tobacco smoking	-0.002	**0.771**	-0.001	-0.088	-0.048	-0.041	0.006	-0.093	-0.049	-0.067	-0.131
	S_I10 Cannabis use	-0.038	**0.878**	-0.031	0.010	-0.058	0.024	0.047	0.006	-0.021	-0.011	0.090
	S_I11 Other drug use	0.005	**0.617**	-0.115	0.164	0.054	-0.114	-0.140	0.019	0.016	0.030	0.065
											
Physical activity												
	AF_I12 Exercise 30 min/day	0.006	-0.068	-0.048	0.019	-0.039	-0.111	0.007	0.023	**0.657**	-0.028	-0.221
	AF_I13 Play 2 h team sports	0.018	-0.108	0.000	-0.119	0.014	-0.003	0.033	-0.048	**0.524**	0.133	0.092
	AF_I14 Choose climb stairs	0.135	-0.067	0.117	-0.035	-0.081	0.003	-0.257	-0.076	0.141	-0.085	-0.038
	AF_I15 Feel good exercising	0.234	0.038	-0.036	-0.094	0.070	-0.063	-0.133	-0.052	**0.480**	-0.093	-0.083
											
Stress management												
	E_I16 Make time to relax	0.039	0.037	0.033	0.108	**-0.416**	0.034	-0.063	-0.033	**0.337**	0.109	0.196
	E_I17 Use cognitive/psychological strategies	0.047	0.003	-0.049	**0.475**	-0.119	0.051	0.026	-0.063	0.052	-0.038	-0.082
	E_I18 Use physical strategies	-0.068	0.008	-0.023	0.289	-0.013	0.120	-0.007	-0.082	**0.670**	-0.011	-0.110
	E_I19 Practice a faith/religion	**0.395**	0.157	0.174	0.085	-0.015	0.075	0.164	-0.032	-0.045	0.154	0.054
	E_I20 Feel good work-life balance	**0.437**	-0.076	0.048	-0.091	-0.183	-0.117	-0.083	-0.082	0.084	0.010	-0.098
	E_I21 Feel work never ends	0.050	0.034	-0.090	**-0.416**	-0.262	0.167	-0.047	-0.082	0.086	-0.077	0.136
	E_I23 Feel life has meaning	**0.728**	-0.013	-0.107	-0.091	-0.058	-0.014	0.018	-0.028	0.018	-0.030	0.010
	E_I24 Feel grateful for the life	**0.714**	0.013	-0.162	-0.015	-0.005	0.004	-0.032	-0.102	0.023	0.027	0.040
											
Sleep												
	S_I25 Sleep 7-9 h/day	-0.046	-0.006	-0.052	-0.010	**-0.789**	-0.047	0.053	-0.034	-0.074	0.042	0.006
	S_I26 Feel rested after sleep	0.132	-0.029	-0.088	-0.108	**-0.539**	-0.010	-0.051	-0.137	0.029	0.002	0.061
	S_I28 Maintain sleep schedule	0.056	0.032	0.031	0.038	**-0.705**	0.012	-0.057	0.053	-0.034	0.001	-0.199
	S_I29 Use sleeping pills	0.096	-0.019	-0.014	**-0.565**	-0.049	0.009	-0.067	0.050	-0.028	0.074	-0.135
											
Social												
	SS_I30 Interact with friend/fam	0.026	0.028	-0.184	0.049	-0.104	0.003	-0.013	-0.056	0.052	**0.555**	0.065
	SS_I31 Belonging	0.263	-0.112	-0.121	0.024	0.012	-0.075	-0.058	-0.074	0.129	**0.404**	0.005
	SS_I32 Has someone to trust	0.166	0.038	**-0.594**	0.000	-0.104	0.046	-0.003	-0.034	-0.025	0.152	-0.178
	SS_I33 Someone helps chores	-0.033	0.061	0.015	-0.118	0.033	-0.105	-0.010	-0.038	-0.002	**0.531**	-0.077
	SS_I34 Has someone to go out	0.102	-0.042	**-0.488**	0.022	-0.019	-0.068	-0.008	0.052	0.157	**0.408**	0.023
	SS_I35 Make yourself available	0.163	-0.062	-0.146	-0.157	-0.007	0.041	0.029	-0.168	0.163	**0.312**	0.029
	SS_I36 Feel loved	**0.338**	0.047	**-0.396**	0.040	-0.013	0.019	-0.069	0.038	0.065	0.238	-0.093
											
Environmental exposure												
	A_I37 More 2 h watching TV	0.016	-0.111	-0.047	0.157	0.073	**0.385**	-0.168	0.032	-0.205	-0.014	-0.099
	A_I38 Smartphone before sleep	-0.032	-0.009	0.003	-0.080	0.012	**0.746**	0.032	-0.064	0.058	-0.054	-0.018
	A_I39 In touch with nature	-0.106	0.028	-0.015	0.030	-0.101	0.025	-0.018	**-0.633**	0.081	0.113	0.022
	A_I40 Feel nature is part of you	0.142	-0.019	0.080	0.049	0.109	0.013	-0.041	**-0.802**	-0.088	0.023	-0.103

EFA = exploratory factor analysis; SMILE = Short Multidimensional Inventory on Lifestyle Evaluation.

Extraction method: principal axis factoring (PAF); rotation method: Oblimin with Kaiser normalization. Bold values represent the loadings (weights) of each variable on the factors.

Subsequent models were performed including the remaining 32 items until reaching the most parsimonious model that presented acceptable goodness-of-fit and internal consistency (Table 2). The final model (Supplementary Material S3) comprised an eight-factor scale with 24 items. The U-SMILE versions in English, Portuguese, and Spanish are presented in Supplementary Material S4.

**Table 2:** Results from the final EFA – U-SMILE (n = 369), Brazil, 2022

		Factor							
Domain/Item		1	2	3	4	5	6	7	8
Diet									
	D_I2 Check labels	0.007	0.060	0.202	-0.001	0.257	0.001	0.006	**-0.455**
	D_I3 Eat processed food	0.081	0.238	**0.364**	-0.068	0.093	-0.049	-0.358	0.158
	D_I5 Eat healthy foods	0.037	0.068	0.074	-0.077	0.206	-0.154	-0.019	**-0.415**
								
Substance use									
	S_I8 Binge drinking	-0.049	**0.550**	0.218	0.004	0.067	0.157	0.327	0.040
	S_I9 Tobacco smoking	-0.117	**0.772**	-0.045	-0.072	0.018	-0.061	0.066	-0.071
	S_I10 Cannabis use	-0.045	**0.822**	-0.114	-0.024	-0.085	-0.015	0.071	0.010
	S_I11 Other drug use	0.149	**0.671**	-0.095	0.092	-0.060	-0.018	-0.160	0.006
								
Physical activity									
	AF_I12 Exercise 30 min/day	0.030	-0.083	-0.142	-0.086	**0.679**	0.051	-0.079	-0.164
	AF_I13 Play 2 h team sports	0.106	-0.135	-0.117	-0.016	**0.444**	-0.108	0.040	0.098
	AF_15 Feel good exercising	0.053	0.055	0.036	-0.023	**0.603**	-0.065	-0.014	0.046
								
Stress management									
	E_I17 Use cognitive/psychological strategies	0.054	-0.023	-0.017	-0.043	-0.111	-0.080	0.005	**-0.318**
	E_I19 Practice a faith or religion	0.139	0.126	0.007	-0.052	-0.037	-0.063	**0.515**	0.032
								
Restorative sleep									
	S_I25 Sleep 7-9 h/day	-0.022	-0.039	-0.086	**-0.932**	-0.096	0.030	-0.007	0.035
	S_I26 Feel rested after sleep	0.094	-0.028	0.033	**-0.588**	0.081	-0.115	-0.017	0.094
	S_I28 Maintain sleep schedule	0.011	0.049	0.072	**-0.639**	0.058	0.063	0.022	-0.137
								
Social support									
	SS_I30 Interact with friends/Family	**0.620**	0.051	-0.044	-0.100	-0.093	-0.062	0.087	0.029
	SS_I31 Belonging	**0.543**	-0.062	-0.001	-0.057	0.114	-0.082	0.112	0.055
	SS_I32 Has someone to trust	**0.619**	0.033	0.047	-0.035	-0.010	0.022	-0.071	-0.232
	SS_I34 Has someone to go out	**0.773**	-0.036	-0.059	0.013	0.049	0.056	-0.052	-0.022
	SS_I35 Make yourself available	**0.475**	-0.060	0.050	-0.025	0.191	-0.141	0.155	0.116
								
Environmental exposure									
	A_I37 More than 2 h watching TV	0.022	-0.102	**0.548**	0.031	-0.161	0.029	-0.063	-0.061
	A_I38 Use cell phone before sleep	-0.099	-0.075	**0.493**	-0.049	0.015	-0.104	0.102	-0.066
	A_I39 In touch with nature	0.004	0.029	-0.064	-0.074	0.003	**-0.722**	-0.087	-0.035
	A_I40 Feel nature is part of you	0.010	0.006	0.113	0.060	0.013	**-0.728**	0.105	-0070

EFA = exploratory factor analysis; SMILE = Short Multidimensional Inventory on Lifestyle Evaluation.

Model fit: root mean square error of approximation (RMSEA) = 0.034, Tucker-Lewis index (TLI) = 0.945, Bayesian information criterion (BIC) = −499.7. Loads with the same color were in the same factor. Bold values represent the loadings (weights) of each variable on the factors.

The U-SMILE presented acceptable internal consistency (Cronbach's α = 0.73; McDonald's ω = 0.79), as well as evidence of concurrent validity (high correlation with the original SMILE), and convergent validity (moderate correlation with PHQ-9 and GAD-7) (Table 3).

**Table 3:** Internal consistency, concurrent validity, and convergent validity of the SMILE* – Solution 1 and the U-SMILE

		Original SMILE* (40 items)	U-SMILE (24 items)
Internal consistency			
	Cronbach's α	0.86	0.73
	McDonald's ω	0.87	0.79
		
Concurrent validity			
	Spearman's r with SMILE*	1	0.94†
	Spearman's r with PHQ-9	-0.553†	-0.517†
	Spearman's r with GAD-7	-0.408†	-0.356†
	Spearman's r with BMI	-0.033	-0.032

BMI = body mass index; GAD-7 = Generalized Anxiety Disorder Questionnaire; PHQ-9 = Patient Health Questionnaire; SMILE = Short Multidimensional Inventory on Lifestyle Evaluation.

* Original SMILE without non-specific and redundant items.

† p < 0.05.

Descriptive statistics of the U-SMILE by sex, age, depression, anxiety, and BMI are presented in Table 4. Individuals without depression or anxiety presented a better lifestyle (i.e., higher U-SMILE scores) as compared with individuals presenting depression and anxiety, respectively. Lifestyle score was better among individuals with low/normal weight as compared with those with obesity; but the difference was not statistically significant.

**Table 4:** U-SMILE* scores by selected sample characteristics (n = 369), Brazil, 2022

Variables		n (%)	Mean (SD)	Median (IRQ)	p-value†
Sex					
	Women	217 (58.8)	67.1 (8.2)	68.0 (11.0)	0.256
	Men	152 (41.2)	68.3 (8.1)	69.0 (10.0)	
				
Age (years)					
	Up to 21	223 (60.4)	67.7 (8.5)	69.0 (10.0)	0.638
	21 +	140 (37.9)	67.4 (7.7)	68.0 (11.0)	
				
PHQ-9‡					
	Negative	201 (54.5)	70.8 (6.9)	71.0 (9.0)	< 0.001
	Positive	128 (34.7)	63.4 (8.0)	63.5 (12.0)	
				
GAD-7‡					
	Negative	171 (46.3)	68.9 (7.4)	69.0 (9.0)	< 0.001
	Positive	158 (42.8)	63.9 (8.0)	64.0 (13.0)	
				
BMI§					
	Normal	263 (71.3)	67.7 (8.1)	69.0 (10.0)	0.487
	Obesity	34 (9.2)	68.0 (7.8)	68.0 (11.0)	

BMI = body mass index; GAD-7 = Generalized Anxiety Disorder Questionnaire; IRQ = interquartile range; PHQ-9 = Patient Health Questionnaire; SD = standard deviation; SMILE = Short Multidimensional Inventory on Lifestyle Evaluation.

* The higher the score, the better the lifestyle.

† p-value independent samples Mann-Whitney test.

‡ Cutoff positive ≥ 10.

§ World Health Organization (WHO) criteria.

## Discussion

In this paper, we presented the shorter version of the SMILE, aimed to evaluate lifestyle among university students, the U-SMILE. The U-SMILE comprised 24 items and had acceptable internal consistency, as well as evidence of convergent and concurrent validity. Improving lifestyle has been shown effective for primary, secondary, and tertiary prevention of mental health disorders.^14,32-34^ Therefore, the correlation between the U-SMILE and the mental health scores was expected and provides evidence of convergent validity. Additionally, our study showed a moderate correlation between lifestyle and mental health measures, and the lack of a strong correlation between these measures indicates that it is unlikely the U-SMILE to be a surrogate measure of depression and/or anxiety (i.e., the U-SMILE is measuring a different construct).

It was expected that U-SMILE score to be correlated with BMI because unhealthy diet and physical inactivity are the major drivers of overweight/obesity.^35^ Herein, we did find that individuals with normal BMI presented better lifestyle scores than those with obesity, but the difference was not statistically significant at 5%. It is possible that the small sample size (regarding the number of obese individuals) has limited the statistical power to detect an association between obesity and the U-SMILE among university students. It is also possible that individuals with higher BMI are trying to change their lifestyle (i.e., adopting a healthier diet and/or exercising, decreasing sedentary behavior) to lose weight, and longitudinal studies will be necessary to disentangle reverse causality.^36^

To reach the U-SMILE, we used a theory-driven approach where the statistical solutions were interpreted following a hypothetical nomological network. In our hypothesis, the lifestyle construct presented seven domains that were correlated with each other. However, our best solution was an eight-factor scale where some items loaded on different factors than expected, e.g., eating processed food loading in the same factor of screen time. There is evidence that high screen time increases the odds of eating processed/unhealthy food in youth,^37,38^ and these behaviors may be correlated (at least moderately).^39^ On the contrary, we expected that screen time and contact with nature were in the same factor, given that the increase in screen time parallels a reduction in time spent in natural environments in recent times.^40^ However, such hypothesis was not confirmed. For these reasons, we recommend the U-SMILE is not used for evaluating domains isolatedly, instead, researchers should consider the overall scores as the main index following the assumption that lifestyle is a single, multidimensional construct.

During the analytical process, we also found that some of the original items were reflecting a different construct. The items in the first model's Factor 1 (i.e., "you feel… good work-life balance," "… feel life has a meaning?," "… feel grateful," and "… feel loved") are likely to be related with well-being instead of lifestyle. It is important to note that the definitions of both, lifestyle and well-being, are matters of controversy and not at all research instruments make it clear the rational/theoretical definitions that underlie item creation/selection. For instance, Linton et al.^41^ found 99 questionnaires for measuring well-being, and many of them included items on alcohol use, social support and physical activity (understood here as lifestyle behaviors). Despite the lack of consensus on the definition of well-being, it can be described as "a state of positive feelings and meeting full potential in the world."^42^ For instance. "Feeling loved" is one item of the Warwick-Edinburgh Mental Well-being Scale (WEMWBS), one of the most used questionnaires for measuring well-being.^43,44^ There is evidence that well-being is associated with healthy behaviors, mental and physical health.^45,46^ We found that the exclusion of the "well-being" items resulted in the subsequent exclusion of another item that could be reflecting mental well-being or emotional eating behavior ("… eat fast-food when you are stressed or sad?").

This study is not free of limitations. First, as any other self-responded survey, social desirability bias may not be excluded, but it has been suggested that anonymous online questionnaires may an efficient strategy to reduce it.^47^ Second, web surveys are prone to selection bias, and it is possible that individuals interested in lifestyle and mental health are more prone to participate – and the error introduced by this bias remains to be addressed in studies profiting from probability samples.^48^ Third, the U-SMILE was developed and validated considering the present definition of lifestyle, and future developments in the field may yield the need for revisions.

Despite these limitations, this paper is based in findings from 10 Brazilian universities from different states/regions and shows the major decisions taken to reach this reduced version of the SMILE. EFA is a complex, interactive, process that has not always been reported in a reproductible manner.^49^ Although there are efforts to improve transparency,^50-52^ researchers still need to take many decisions that are impossible to publish in scientific papers. Beavers et al.^52^ emphasize the importance of theoretical knowledge and common sense to reach the most "parsimonious, mathematically sound, and theoretically grounded" solution. We add that, among the multiple mathematically sound possible solution, authors should make clear in which way the theory drove the process to reach the final solution.

Finally, we believe that the U-SMILE helps to fill out a gap in improving the measurement of lifestyle, in general, and among university students, which must be an overarching goal for clinical and epidemiological research.
