# Supplementary material for: U-SMILE: a brief version of the Short Multidimensional Inventory on Lifestyle Evaluation
Source: Trends Psychiatry Psychother. 2025 Apr 7;47:e20230722. doi: 10.47626/2237-6089-2023-0722 (PMC12904267; doi:10.47626/2237-6089-2023-0722)
Supplement: Supplementary file 1 [file 2238-0019-trends-47-e20230722-suppl1.pdf]

http://doi.org/10.47626/2237-6089-2023-0722

Supplementary Material S1 - Normality check

Domain	Item	Normality test
Diet and nutrition	D_I1	W = 0.76714, p < 2.2e-16
	D_I2	W = 0.79511, p < 2.2e-16
	D_I3	W = 0.79032, p < 2.2e-16
	D_I4	W = 0.86333, p < 2.2e-16
	D_I5	W = 0.83844, p < 2.2e-16
	D_I6	W = 0.86555, p < 2.2e-16
	D_I7	W = 0.86671, p < 2.2e-16
Substance use	S_I8	W = 0.77855, p < 2.2e-16
	S_I9	W = 0.48816, p < 2.2e-16
	S_I10	W = 0.44103, p < 2.2e-16
	S_I11	W = 0.1617, p < 2.2e-16
Physical activity	AF_I12	W = 0.83066, p < 2.2e-16
	AF_I13	W = 0.77501, p < 2.2e-16
	AF_I14	W = 0.87736, p < 2.2e-16
	AF_I15	W = 0.61632, p < 2.2e-16
Stress management	E_I16	W = 0.86326, p < 2.2e-16
	E_I17	W = 0.74878, p < 2.2e-16
	E_I18	W = 0.83673, p < 2.2e-16
	E_I19	W = 0.83042, p < 2.2e-16
	E_I20	W = 0.87288, p < 2.2e-16
	E_I21	W = 0.85616, p < 2.2e-16
	E_I22	W = 0.8663, p < 2.2e-16
	E_I23	W = 0.85026, p < 2.2e-16
	E_I24	W = 0.79149, p < 2.2e-16
Sleep	S_I25	W = 0.87226, p < 2.2e-16
	S_I26	W = 0.87204, p < 2.2e-16
	S_I27	W = 0.83846, p < 2.2e-16
	S_I28	W = 0.45836, p < 2.2e-16
	S_I29	W = 0.76071, p < 2.2e-16
Social support	SS_I30	W = 0.849, p < 2.2e-16
	SS_I31	W = 0.82161, p < 2.2e-16
	SS_I32	W = 0.80604, p < 2.2e-16
	SS_I33	W = 0.81666, p < 2.2e-16
	SS_I34	W = 0.83188, p < 2.2e-16
	SS_I35	W = 0.78636, p < 2.2e-16
	SS_I36	W = 0.83692, p < 2.2e-16
Environmental exposure	A_I37	W = 0.64923, p < 2.2e-16
	A_I38	W = 0.85397, p < 2.2e-16
	A_I39	W = 0.83136, p < 2.2e-16
	A_I40	W = 0.81666, p < 2.2e-16

Supplementary Material S3

Table S1 - Total variance explained

	Initial eigenvalues			Extraction sums of squared loadings			Rotation sums of squared loadings*
**Factor**	**Total**	**% of variance**	**Cumulative %**	**Total**	**% of variance**	**Cumulative %**	**Total**
1	4.566	19.023	19.023	4.065	16.937	16.937	2.990
2	2.676	11.150	30.173	2.250	9.376	26.313	2.201
3	1.893	7.887	38.060	1.302	5.427	31.740	1.024
4	1.463	6.096	44.156	1.001	4.172	35.912	2.592
5	1.379	5.747	49.903	0.891	3.711	39.623	1.950
6	1.226	5.109	55.012	0.674	2.808	42.431	2.262
7	1.136	4.731	59.744	0.534	2.225	44.656	0.700
8	1.060	4.417	64.161	0.455	1.896	46.552	0.989
9	0.913	3.803	67.964				
10	0.818	3.407	71.370				
11	0.697	2.903	74.274				
12	0.680	2.832	77.105				
13	0.635	2.648	79.753				
14	0.609	2.536	82.289				
15	0.568	2.368	84.657				
16	0.535	2.229	86.886				
17	0.484	2.017	88.903				
18	0.465	1.939	90.842				
19	0.464	1.935	92.777				
20	0.392	1.634	94.412				
21	0.380	1.582	95.993				
22	0.340	1.417	97.410				
23	0.334	1.392	98.802				
24	0.288	1.198	100.000				

Extraction method: principal axis factoring (PAF).
* When factors are correlated, sums of squared loadings cannot be added to obtain a total variance.

Table S2 - KMO and Bartlett's Test

Kaiser-Meyer-Olkin Measure of sampling adequacy	0.778
Bartlett's test of sphericity	
Chi-square Approximation	2346.002
Degrees of freedom (df)	276
Sig.	0.000

Table S3 - Communalities matrix

Items	Initial	Extraction
D_I2C	0.257	0.369
D_I3	0.184	0.369
D_I5C	0.288	0.364
S_I8	0.384	0.518
S_I9	0.508	0.615
S_I10	0.562	0.695
S_I11	0.416	0.486
AF_I12C	0.360	0.559
AF_I13C	0.281	0.325
AF_I15C	0.310	0.422
E_I17C	0.117	0.136
E_I19C	0.202	0.341
S_I25C	0.485	0.786
S_I26C	0.438	0.484
S_I28C	0.409	0.485
SS_I30C	0.405	0.467
SS_I31C	0.402	0.439
SS_I32C	0.377	0.468
SS_I34C	0.464	0.598
SS_I35C	0.378	0.443
A_I37	0.220	0.336
A_I38	0.223	0.308
A_I39C	0.399	0.565
A_I40C	0.415	0.594

Extraction method: principal axis factoring (PAF).

Table S4 - Factor correlation matrix

Factor	1	2	3	4	5	6	7	8
1	1.000	-0.028	0.079	**0.370**	**0.295**	**0.399**	0.028	0.073
2	-0.028	1.000	**0.106**	-0.049	0.033	0.033	-0.061	0.028
3	0.079	**0.106**	1.000	-0.080	-0.037	-0.113	0.040	-0.191
4	**0.370**	-0.049	-0.080	1.000	**0.247**	**0.348**	0.090	**0.200**
5	**0.295**	0.033	-0.037	**0.247**	1.000	**0.249**	-0.011	0.051
6	**0.399**	0.033	-0.113	**0.348**	**0.249**	1.000	**0.091**	**0.210**
7	0.028	-0.061	0.040	0.090	-0.011	**0.091**	1.000	-0.003
8	0.073	0.028	-0.191	0.200	0.051	**0.210**	-0.003	1.000

Extraction method: principal axis factoring (PAF); rotation method: Oblimin with Kaiser normalization. The bold values represent the loadings (weights) of each variable on the factors.

Supplementary Material S4

English version – U-SMILE: University Short Multidimensional Inventory Lifestyle Evaluation

	In the last month, how often in your daily routine…	**Always**	**Often**	**Seldom**	**Never**
Diet and nutrition	1. When shopping for food, do you check labels for ingredients such as quantity of salt?	4	3	2	1
	2. Do you eat processed food (frozen food such as pizza, French fries, puff pastries, deep-fried foods and canned foods)?	1	2	3	4
	3. Do you eat healthy foods such as fresh fruits, fresh vegetables, wholegrain, legumes or nuts?	4	3	2	1
Substance use	4. Do you drink 5 or more doses (men) or 4 or more doses (women) of alcoholic beverages on a single occasion, which means within 2 hours? (1 dose of alcohol=1 glass of beer OR 1 glass of wine OR 1 shot of spirit (such as rum, vodka, whisky, tequila or gin)).	1	2	3	4
	5. Do you smoke tobacco (cigarette, electronic cigarette, cigar, pipe, smokeless tobacco)?	1	2	3	4
	6. Do you use marijuana or hashish?	1	2	3	4
	7. Do you use other drugs (cocaine, crack, amphetamines, ecstasy, opioids without medical prescription, and others)?	1	2	3	4
Physical activity	8. Do you exercise for at least 30 minutes daily (or 150 minutes a week)?	4	3	2	1
	9. Do you play at least 2 hours of team sports (like soccer, volleyball, basketball, rugby, etc.) a week?	4	3	2	1
	10. Do you feel good after performing physical activity?	4	3	2	1
Stress management	11. Do you use any strategy or psychological support to deal with stress (for instance meditation, mindfulness or psychotherapy)?	4	3	2	1
	12. Do you practice a faith or religion?	4	3	2	1
Sleep	13. Do you manage to sleep between 7 and 9 hours per night?	4	3	2	1
	14. Do you feel rested with the number of hours you sleep?	4	3	2	1
	15. Do you maintain a regular sleep schedule?	4	3	2	1
Social support	16. Do you interact with your friends and/or relatives?	4	3	2	1
	17. Do you feel that you are part of a group of friends, the community or the society?	4	3	2	1
	18. Do you have someone you trust who listens to your problems or concerns?	4	3	2	1
	19. Do you have someone in your life to go out or have fun with when you feel like it?	4	3	2	1
	20. Do you make yourself available to support your significant ones?	4	3	2	1
Environment exposures	21. Do you spend more than 2 hours a day watching TV, playing computer games, video games or on the internet?	1	2	3	4
	22. Do you spend time on a computer/smartphone within 1 hour of going to sleep?	1	2	3	4
	23. Are you in touch with nature (for instance parks, beach, countryside, mountains)?	4	3	2	1
	24. Do you feel your relationship to nature, that is all living things, is an important part of who you are?	4	3	2	1

The score is provided by summing up all the answers.

Versão em Português – U-SMILE: Breve Inventário para Avaliação Multidimensional do Estilo de Vida de Universitários

	**No último mês,** com que frequência na sua rotina diária, você…	**Sempre**	**Frequentemente**	**Eventualmente**	**Nunca**
Dieta e Nutrição	1. Ao comprar comida, verificou os rótulos quanto a ingredientes, como quantidade de sal?	4	3	2	1
	2. Consumiu alimentos pré-prontos (congelados tais como pizza, batata-frita, empanados em geral e enlatados)?	1	2	3	4
	3. Comeu alimentos saudáveis, tais como frutas e vegetais frescos, legumes, produtos integrais ou amendoim, nozes, castanhas, etc.?	4	3	2	1
Uso de substâncias	4. Bebeu 5 ou mais doses de bebidas alcoólicas* (homem) ou 4 ou mais doses (mulher) em uma única ocasião, ou seja, em cerca de 2hs?	1	2	3	4
	5. Utilizou derivados do tabaco (cigarro, cigarro eletrônico, charuto, cachimbo, fumo de corda)?	1	2	3	4
	6. Utilizou maconha, Skank, haxixe?	1	2	3	4
	7. Utilizou outras drogas ilícitas (cocaína, crack, anfetaminas, ecstasy, opioides sem prescrição médica, etc.)?	1	2	3	4
Atividade Física	8. Se exercitou pelo menos 30 minutos/dia (ou 150 minutos por semana)?	4	3	2	1
	9. Praticou pelo menos 2 horas de esporte coletivo (futebol, voleibol, basquete) por semana?	4	3	2	1
	10. Sentiu-se bem após realizar atividade física?	4	3	2	1
Gerenciar stress	11. Usou de estratégias cognitivas OU suporte psicológico para lidar com o estresse (por exemplo: meditação, mindfulness e psicoterapia)?	4	3	2	1
	12. Praticou uma crença, religião ou espiritualidade?	4	3	2	1
Sono	13. Dormiu entre 7 e 9 horas por dia?	4	3	2	1
	14. Sentiu-se descansado(a) com o número de horas dormidas?	4	3	2	1
	15. Manteve a regularidade em relação aos horários de sono?	4	3	2	1
Suporte Social	16. Interagiu com seus amigos e/ou familiares?	4	3	2	1
	17. Teve a sensação de pertencimento OU sentiu-se incluído (sentiu que faz parte de um grupo de amigos, de uma comunidade, da sociedade)?	4	3	2	1
	18. Teve alguém de confiança para escutar seus problemas/preocupações?	4	3	2	1
	19. Teve companhia para sair/disfrutar OU curtir/divertir quando necessitou?	4	3	2	1
	20. Esteve disponível para pessoas importantes para você?	4	3	2	1
Ambiente	21. Gastou mais de 2 horas por dia assistindo TV, jogando jogos de computador, videogames ou navegando na internet?	1	2	3	4
	22. Ficou no computador ou smartphone na hora imediatamente anterior a ir dormir?	1	2	3	4
	23. Esteve em contato com a natureza (parques, praia, campo, montanha)?	4	3	2	1
	24. Sentiu que seu relacionamento com a natureza, com todas as coisas vivas, é uma parte importante de quem você é?	4	3	2	1

O escore da U-SMILE é calculado pela soma de todas as respostas.

http://doi.org/10.47626/2237-6089-2023-0722

Versión en español – U-SMILE: Breve Inventario para la Evaluación Multidimensional del Estilo de Vida de los Universitarios

	En su rutina diaria durante el último mes, ¿con qué frecuencia usted…?	Siempre	Con frecuencia	A veces	Nunca
Dieta y Nutrición	1. Cuando ha hecho la compra, se ha fijado en las etiquetas de ingredientes de los alimentos, como la cantidad de sal	4	3	2	1
	2. Ha comido alimentos procesados, es decir, congelados como pizzas, patatas fritas, bollería industrial, fritos y comida enlatada	1	2	3	4
	3. Ha comido alimentos saludables como frutas frescas, verduras frescas, cereales integrales, legumbres o frutos secos	4	3	2	1
Consumo de sustancias	4. Ha bebido 5 o más dosis (si es hombre) o 4 o más dosis (si es mujer) de bebidas alcohólicas* en una sola ocasión, es decir, en menos de 2 horas	1	2	3	4
	5. Ha fumado tabaco (cigarrillos, cigarrillo electrónico, puro, pipa, tabaco sin humo	1	2	3	4
	6. Ha fumado marihuana o hachís	1	2	3	4
	7. Ha tomado otras drogas (cocaína, crack, anfetaminas, éxtasis, opiáceos sin receta médica u otras drogas)	1	2	3	4
Actividad física	8. Ha realizado ejercicio físico al menos 30 minutos cada día (o 150 minutos a la semana)	4	3	2	1
	9. Ha hecho deporte de equipo (fútbol, voleibol, baloncesto, rugby…) al menos 2 horas a la semana	4	3	2	1
	10. Se ha sentido bien después de hacer actividades físicas	4	3	2	1
Manejar el estrés	11. Ha usado alguna estrategia o apoyo de tipo psicológico para manejar el estrés. Por ejemplo, meditación, mindfulness o psicoterapia	4	3	2	1
	12. Ha practicado alguna religión o creencia espiritual	4	3	2	1
Dormir	13. Ha logrado dormir entre 7 y 9 horas cada noche	4	3	2	1
	14. Ha sentido que ha descansado con el número de horas que duerme	4	3	2	1
	15. Ha mantenido un horario de sueño regular	4	3	2	1
Apoyo social	16. Ha interactuado con sus amigos y/o familiares	4	3	2	1
	17. Ha sentido que forma parte de un grupo de amigos, de su comunidad o de la sociedad	4	3	2	1
	18. Ha tenido alguien de su confianza disponible que escuche sus problemas o preocupaciones	4	3	2	1
	19. Ha tenido a alguien en su vida con quien salir o divertirse cuando le apetece	4	3	2	1
	20 . Ha estado disponible para ayudar a sus seres queridos	4	3	2	1
Ambiente	21. Ha pasado más de 2 horas al día viendo la televisión, jugando en el ordenador, videojuegos o en internet	1	2	3	4
	22. Ha usado el ordenador o el móvil 1 hora antes de acostarse	1	2	3	4
	23. Ha dedicado tiempo a estar en contacto con la naturaleza, por ejemplo en parques, la playa, el campo o la montaña	4	3	2	1
	24. Ha sentido que su relación con la naturaleza, es decir todos los seres vivos, es una parte importante de quién es usted	4	3	2	1

La puntuación de U-SMILE se calcula sumando todas las respuestas.
